# Systemic Complement Activation Profiles in Nonexudative Age-Related Macular Degeneration: A Meta-Analysis

**DOI:** 10.3390/jcm11092371

**Published:** 2022-04-23

**Authors:** Jonathan B. Lin, Stylianos Serghiou, Joan W. Miller, Demetrios G. Vavvas

**Affiliations:** 1 Department of Ophthalmology, Mass Eye and Ear and Harvard Medical School, Boston, MA 02114, USA; jonathan_lin@meei.harvard.edu (J.B.L.); joan_miller@meei.harvard.edu (J.W.M.); 2 Google LLC, Mountain View, CA 94043, USA; stelios.serghiou@gmail.com

**Keywords:** complement, age-related macular degeneration, meta-analysis, geographic atrophy

## Abstract

Although complement inhibition has emerged as a possible therapeutic strategy for age-related macular degeneration (AMD), there is not a clear consensus regarding what aspects of the complement pathway are dysregulated in AMD and when this occurs relative to disease stage. We recently published a systematic review describing systemic complement activation profiles in patients with early/intermediate AMD or geographic atrophy (GA) compared to non-AMD controls. Here, we sought to meta-analyze these results to estimate the magnitude of complement dysregulation in AMD using restricted maximum likelihood estimation. The seven meta-analyzed studies included 710 independent participants with 23 effect sizes. Compared with non-AMD controls, patients with early/intermediate nonexudative AMD (N = 246) had significantly higher systemic complement activation, as quantified by the levels of complement proteins generated by common final pathway activation, and significantly lower systemic complement inhibition. In contrast, there were no statistically significant differences in the systemic levels of complement common final pathway activation products or complement inhibition in patients with GA (N = 178) versus non-AMD controls. We provide evidence that systemic complement over-activation is a feature of early/intermediate nonexudative AMD; no such evidence was identified for patients with GA. These findings provide mechanistic insights and inform future clinical trials.

## 1. Introduction

Age-related macular degeneration (AMD) is a leading cause of blindness among older adults [1]. Advanced AMD can cause devastating vision loss in two forms: advanced exudative AMD, characterized by choroidal neovascularization, and advanced nonexudative AMD, characterized by the death of photoreceptors and the retinal pigment epithelium (RPE), termed geographic atrophy (GA). Therapies that target vascular endothelial growth factor (VEGF) are available for treating patients with advanced exudative AMD. However, there are no approved therapies for patients with advanced nonexudative AMD. Antioxidant and mineral supplementation (AREDS/AREDS2) is sometimes suggested for patients with AMD, but it has not been proven to have an effect in slowing progression to GA and has not been tested in a large, randomized clinical trial [2,3,4,5,6].

Given a strong association between complement dysregulation and AMD from both genetic and preclinical studies as reviewed elsewhere [7,8], some have speculated that complement inhibition may have a therapeutic role in slowing the progression of GA. As a result, there have been significant efforts directed towards testing this possibility in patients with nonexudative AMD. Despite promising preclinical studies, many of these human clinical trials testing complement inhibition have failed to show any significant reduction in the progression of GA, although some agents do remain under active investigation [7,8]. These findings suggest that the role of complement dysregulation in AMD pathogenesis may be complex and warrants further investigation.

We recently performed a systematic review to determine whether there are differences in systemic complement activation profiles in patients with early to intermediate nonexudative AMD and those with GA compared with non-AMD controls [9]. Here, we meta-analyzed the available evidence from these studies identified in our prior systematic review to estimate the magnitude of complement overactivation in patients with early/intermediate AMD and in those with GA. These findings not only will improve our understanding of the molecular pathogenesis of AMD but also may enable us to identify the appropriate patient population for complement-based therapies.

## 2. Materials and Methods

### 2.1. Protocol and Registration

The review protocol was not registered prior to publication. We followed the Preferred Reporting Items for Systematic Reviews and Meta-Analyses (PRISMA) [10] and Meta-analysis of Observational Studies in Epidemiology (MOOSE) [11] guidelines.

### 2.2. Search Strategy and Study Selection

We meta-analyzed the eight articles identified in our prior systematic review. The comprehensive literature search strategy was described previously [9]. Restated here, we performed a comprehensive literature search using PubMed, Embase, and Google Scholar on 11 October 2020. The full electronic search syntax used in PubMed was: *macular degeneration* [MESH terms] OR *age related macular degeneration* [All fields] OR *AMD* [All fields] AND *complement* [All fields]; the syntax used in Embase was: (‘age related macular degeneration’/exp OR ‘age related macular degeneration’ OR ‘amd’/exp OR amd OR ‘macular degeneration’/exp OR ‘macular degeneration’) AND (‘complement’/exp OR complement) AND ‘article’/it; the syntax used in Google Scholar was: allintitle: “age related macular degeneration” OR AMD OR “macular degeneration” AND complement. For all three databases, we had no restrictions based on publication date or article type. This search identified a total of 1627 manuscripts after duplicates were removed. J.B.L. reviewed the title and abstracts of each of these manuscripts, omitting those written in a language other than English, those clearly not relevant to the present study, and review articles. There were no controversial cases that required adjudication. We then obtained the full texts of the remaining studies and assessed them for inclusion. We also reviewed the references of each included article to identify other potential articles for inclusion. This comprehensive literature search was repeated on 7 January 2022 in the same manner and did not identify any additional manuscripts for inclusion.

We included all studies, irrespective of the study design, that reported quantitative values for at least one complement protein and compared either patients with nonexudative AMD by any classification methodology versus non-AMD controls or patients with either central or non-central GA versus non-AMD controls. We did not require the quantification of complement proteins to be the primary aim of the study. Since we were interested in systemic complement activation patterns in nonexudative AMD and in GA, we excluded any study that did not differentiate between AMD subtype or that included multiple types of AMD (exudative AMD and GA) in a single comparison group, as these studies had inappropriate case definition for our research question. Although some of the included studies were performed by the same groups, there was no indication within the full text that they originated from the same specific patients.

### 2.3. Summary Measures and Synthesis of the Results

We performed statistical analysis and data visualization with R 4.0.3 [12] and RStudio Desktop 1.3.1093 (Boston, MA, USA), using the packages *meta* [13] and *metafor* [14]. For the seven included studies, we calculated standardized mean differences (SMD) using Hedges’ *g*. SMD is the difference in means between cases and controls divided by the total standard deviation; for reference, SMD of 0.2 is considered a small difference, SMD of 0.5 is considered a medium difference, and SMD of 0.8 is considered a large difference [15]. Hedges’ *g* is an unbiased estimator of SMD suitable for small sample sizes [16]. For the four (57%) studies that reported medians instead of means [17,18,19,20], we approximated mean and standard deviation from median and range or median and interquartile range, using previously validated formulae [21,22]. J.B.L. performed the data extraction, which was confirmed independently by D.G.V.

Using restricted maximum likelihood (REML) estimation, we generated multi-level random-effects (MLRE) models using the function rma.mv of the *metafor* package and adjusted the estimated standard errors using the Knapp–Hartung correction. Multi-level or hierarchical/nested models were chosen to account for possible intra-study correlations, since we allowed for the inclusion of multiple SMD when multiple complement proteins were reported in a single study. We considered two-tailed *p* < 0.05 to be statistically significant. One study reported 10th and 90th percentiles rather than range or interquartile range [20]. MLRE models generated with estimates of mean/standard deviation yielded similar results whether 10th/90th percentiles were set equal to interquartile range or minimum/maximum; here, we report MLRE models based on the former assumption, since this is a more conservative approach. We also calculated 95% prediction intervals to show the range of effect sizes we would expect to observe in future studies after accounting for the differences observed between the meta-analyzed studies included here.

### 2.4. Assessment of Heterogeneity, Outliers, and Publication Bias

We assessed between-study heterogeneity using Higgin’s and Thompson’s I^2^, which reports a percentage that describes the proportion of uncertainty due to heterogeneity. Heterogeneity is the variability between meta-analyzed studies. High heterogeneity (e.g., I^2^ > 50%) indicates that the effect sizes from the meta-analyzed studies are different enough that there may be two or more subgroups of studies within the data, making it more difficult to interpret the pooled effect size. We defined potentially influential outliers as SMD whose 95% confidence intervals did not overlap with the 95% confidence interval of the pooled standardized mean effect. Because no influential outliers were identified, no additional sensitivity analyses were performed. No test for publication bias was necessary because no more than three studies contributed to each of the meta-analyses.

### 2.5. Data and Code Sharing

The data are openly available under a CC-BY-NC 4.0 license, and the code is available under a GPL 3.0 license on GitHub at: https://github.com/jonathanblin/complement-amd-meta-analysis.

## 3. Results

### 3.1. Study Characteristics

**Figure 1:**
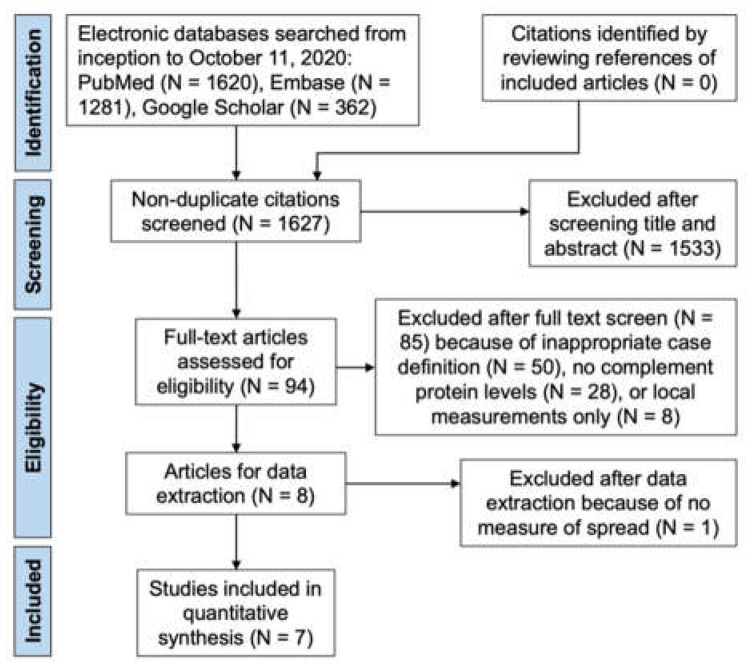
Preferred Reporting Items for Systematic Reviews and Meta-Analyses (PRISMA) Flow Diagram showing citations identified, included, and excluded with reasons for exclusions. Adapted with permission from ref. [9] with modifications. 2022, American Academy of Ophthalmology.

Our prior systematic review identified eight manuscripts for inclusion in this meta-analysis. Of these studies, one study had to be excluded after data extraction, since it did not report any measure of dispersion such as standard deviation or range, which are necessary for a meta-analysis. Figure 1 depicts a flow diagram of how we identified the seven studies meta-analyzed in this manuscript.

In total, the meta-analysis included 710 independent patients from 7 studies [17,18,19,20,23,24,25] (mean per study: 101; standard deviation [SD]: 35) that reported 23 effect sizes of interest (Table 1). Although the 7 included studies had a cumulative total of 920 patients, 210 patients with exudative AMD from 4 studies were omitted from the final analysis. Two studies (29%) were cross-sectional; two (29%) were hybrid studies that combined data of patients from the observational (pre-treatment) phase of a phase 2 randomized clinical trial and a separate cross-sectional study; and three (43%) were case–control studies that used a subset of patients from AMD registries. The majority (71%) of the studies originated from the United States. The included studies used different AMD classification criteria, including those from the International ARM Epidemiological Study Group, the Clinical Age-Related Maculopathy Staging (CARMS) system, the Age-Related Eye Disease Study (AREDS) severity scale, or investigator-defined clinical criteria (Table 1).

To facilitate the meta-analysis, we grouped the complement proteins into those that are generated during common final pathway activation (C3a, C3a-desArg, C5a, Ba, Bb, sC5b-9) and those that either inhibit complement activation or are inactivated complement products (CFH, CFI, iC3b). This approach was chosen a priori. Although some of the included studies performed multivariable analysis to control for covariates such as age and gender, we calculated standardized mean differences based on unadjusted values to permit the meta-analysis of all included studies, including those that performed solely univariable analysis. There were notable differences between the studies with respect to the specific complement proteins measured (Table 2).

### 3.2. Quality Assessment

Using a modified Newcastle–Ottawa (NOS) scale, we previously found all meta-analyzed studies had a low risk of bias, given NOS scores greater than or equal to 7 [9].

### 3.3. Complement Common Pathway Activation

**Figure 2:**
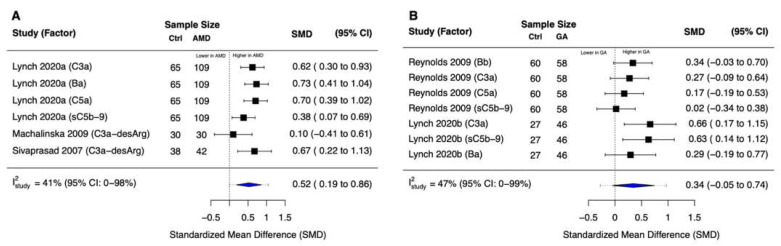
Forest plots for common final pathway activation products. (**A**) We observed significantly higher systemic levels of complement common final pathway activation products in patients with early to intermediate nonexudative AMD versus non-AMD controls (*p* = 0.01). (**B**) There was no statistically significant difference in the systemic levels of complement common final pathway activation products in patients with geographic atrophy versus non-AMD controls (*p* = 0.08). Blue diamonds denote the 95% confidence interval (CI); dashed tails denote the 95% prediction interval for this multi-level random-effects model.

By meta-analyzing six effect sizes from three studies, we found that patients with early to intermediate nonexudative AMD (N = 181) had significantly higher systemic levels of complement common final pathway activation products compared with control patients (standardized mean difference [SMD] = 0.52; 95% confidence interval [CI]: 0.19 to 0.86) (Figure 2A). There was moderate heterogeneity in the meta-analyzed effect sizes (between-study I^2^ [I^2^_study_] = 41%; 95% CI: 0% to 98%), but it was not to a prohibitive level that would make it difficult to interpret the pooled effect. In contrast, the meta-analysis of seven effect sizes from two studies revealed that the difference in the systemic levels of complement common final pathway activation products in patients with GA (N = 104) versus control patients was not statistically significant (SMD = 0.34 [95% CI: −0.05 to 0.74]), with moderate heterogeneity (I^2^_study_ = 47% [95% CI: 0% to 99%]) (Figure 2B).

### 3.4. Complement Inhibition

**Figure 3:**
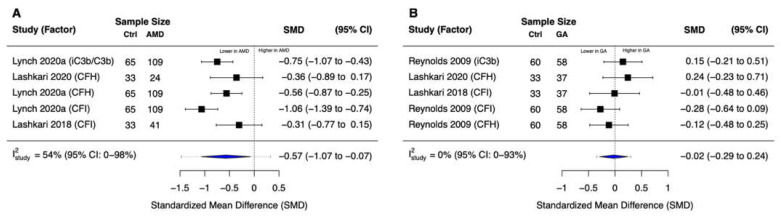
Forest plots for complement inhibition. (**A**) We observed statistically significantly lower systemic complement inhibition in patients with early to intermediate nonexudative AMD versus non-AMD controls (*p* = 0.03). (**B**) There was no statistically significant difference in systemic complement inhibition in patients with geographic atrophy versus non-AMD controls (*p* = 0.82). Blue diamonds denote the 95% confidence interval (CI); dashed tails denote the 95% prediction interval for this multi-level random-effects model.

The meta-analysis of five effect sizes from three studies revealed that patients with nonexudative AMD (N = 174) had significantly lower systemic complement inhibition compared with control patients (SMD = −0.57 [95% CI: −1.07 to −0.07]; I^2^_study_ = 54% [95% CI: 0% to 98%]) (Figure 3A). In contrast, based on five effect sizes from three studies, there was no statistically significant difference in systemic complement inhibition in patients with GA (N = 132) versus control patients (SMD = −0.02 [95% CI: −0.29 to 0.24]; I^2^_study_ = 0% [95% CI: 0% to 93%]) (Figure 3B).

### 3.5. Risk of Influential Outliers

We did not detect influential outliers and thus did not perform a sensitivity analysis.

## 4. Discussion

In this meta-analysis, we gathered all published studies to date comparing the systemic levels of complement proteins in patients with early to intermediate nonexudative AMD or GA versus non-AMD controls. Despite a substantial heterogeneity between the synthesized studies in terms of study design, AMD case definition, and the specific complement proteins measured, our findings suggest increased systemic complement common final pathway activation and decreased systemic complement inhibition in patients with nonexudative AMD versus non-AMD controls. No significant differences were found in systemic complement common final pathway activation and systemic complement inhibition in patients with GA versus non-AMD controls.

These findings suggest that complement pathway over-activation may be involved in the pathogenesis of early or intermediate stages of nonexudative AMD. While our results are inconclusive about GA, the small effect sizes suggest that complement over-activation may be less important in this disease stage. These findings may explain why some Phase 3 clinical trials examining the utility of complement inhibition strategies for slowing GA progression have failed to find a significant result. If complement over-activation is indeed a more prominent feature of earlier rather than advanced disease, complement inhibition strategies may be better suited for targeting patients with earlier forms of the disease. Further studies should also examine whether alterations in systemic complement activation profiles also reflect local, intraocular complement activity. While alterations in complement activation profiles in aqueous humor have been reported in patients with early to intermediate AMD, whether this shows a concordant pattern systemically remains unexplored [26,27].

Our study has several limitations. First, there were fewer patients with GA (N = 178) than patients with nonexudative AMD without GA (N = 246) that were available to be meta-analyzed, which could explain the absence of significance in patients with GA on the basis of reduced statistical power. We addressed this limitation by combining results within and between studies using a multivariable meta-analysis. Addition of future studies to this meta-analysis may provide the larger sample size needed for more conclusive results. Second, there was notable heterogeneity between the meta-analyzed studies in terms of AMD case definition, specific complement proteins quantified, selection of controls, and country of study origin. We quantified this between-study variation with I^2^, which did not suggest prohibitive heterogeneity, and included prediction intervals in our forest plots to illustrate this variation. Finally, four studies reported median and range/interquartile range, which we converted into mean and standard deviation using established formulae. Even though these methods have been validated by other groups [21,22], this methodology may have introduced bias.

## 5. Conclusions

Despite these limitations, our findings provide evidence that systemic complement over-activation is present in early to intermediate nonexudative AMD, whereas no evidence that complement over-activation is present in GA was found. These findings provide a foundation for further mechanistic studies investigating the utility of complement pathway modulation in nonexudative AMD and GA and may inform the design of future clinical trials to uncover the utility of complement inhibition for treating AMD. Furthermore, additional studies that examine local complement activation status in different stages of AMD are needed.

## Figures and Tables

**Table 1:** Characteristics of the Studies Included in the Meta-Analysis.

Study (Country)	Year	Study Design	Number of Participants			Controls	AMD Classification Methodology
			neAMD ^a^	GA	Non-AMD		
Sivaprasad et al. (England)	2007	Cross-sectional	42	0	38	Likely clinic-based; healthy without AMD	International ARM Epidemiological Study Group
Reynolds et al. (USA)	2009	Case-control from registry	0	58	60	Registry-based; no AMD, CARMS grade 1	Clinical Age-Related Maculopathy Staging
Machalińska et al. (Poland)	2009	Cross-sectional	30	0	30	Clinic-based; no AMD	Study-specific clinical definition
Lashkari et al. (USA)	2018	Hybrid	41	37	33	Clinic-based; no AMD, AREDS stage 0	Age-Related Eye Disease Study
Lynch et al. (USA) ref. [18]	2020b	Case-control from registry	0	46	27	Registry-based; cataract controls without AMD	Study-specific clinical definition
Lynch et al. (USA) ref. [17]	2020a	Case-control form registry	109	0	65	Registry-based; cataract controls without AMD	Study-specific clinical definition
Lashkari et al. (USA)	2020	Hybrid	24	37	33	Clinic-based; no AMD, AREDS stage 0	Age-Related Eye Disease Study

^a^ Nonexudative age-related macular degeneration.

**Table 2:** Complement Proteins Measured in Each Meta-Analyzed Study.

Study	Year	Activation Products						Inhibition		
		C3a	C3a-desArg	Ba	Bb	C5a	sC5b-9	CFH	CFI	iC3b/C3b
Sivaprasad et al.	2007		**✗**							
Reynolds et al.	2009	**✗**			**✗**	**✗**	**✗**	**✗**	**✗**	**✗**
Machalińska et al.	2009		**✗**							
Lashkari et al.	2018								**✗**	
Lynch et al. ref. [18]	2020b	**✗**		**✗**			**✗**			
Lynch et al. ref. [17]	2020a	**✗**		**✗**		**✗**	**✗**	**✗**	**✗**	**✗**
Lashkari et al.	2020							**✗**		

## Data Availability

The data are available under a CC-BY-NC 4.0 license, and the code is available under a GPL 3.0 license: https://github.com/jonathanblin/complement-amd-meta-analysis.
